# A bio-coupling approach using a dextran-binding domain to immobilize an engineered streptavidin to Sephadex for easy preparation of affinity matrix

**DOI:** 10.1038/s41598-019-40044-4

**Published:** 2019-03-04

**Authors:** Sau-Ching Wu, Chris Wang, Jonathan Chin, Sui-Lam Wong

**Affiliations:** 1 Department of Biological Sciences, University of Calgary, Calgary, Alberta, Canada; 2 Present Address: Biology Program, Faculty of Arts & Science, Ambrose University, 150 Ambrose Circle SW, Calgary, Alberta T3H 0L5 Canada

## Abstract

An engineered streptavidin, SAVSBPM18 with reversible biotin binding capability, has been successfully applied to purify biotinylated and streptavidin-binding peptide (SBP) tagged proteins. To simplify the preparation for the SAVSBPM18 affinity matrix without chemical conjugation, two bio-coupling approaches were developed based on a 14-kDa dextran-binding domain (DBD) from a *Leuconostoc mesenteroides* dextransucrase. The first approach offers simplicity for bio-coupling by creating a direct fusion, SAVSBPM18-Linker-DBD. Purification of the fusion from crude extract and its immobilization to Sephadex can be consolidated in one-step. The second approach aims at flexibility. A SnoopCatcher (SC) was fused to DBD to create SC-Linker-DBD. This fusion can covalently capture any recombinant proteins tagged with a SnoopTag (ST) including SAVSBPM18-Linker-ST via the formation of an isopeptide bond at the interface through the SnoopCatcher-SnoopTag interaction. Although monomeric DBD binds to dextran with nanomolar affinity, DBD tetramerized via streptavidin (SAVSBPM18-Linker-ST·SC-Linker-DBD) showed an even tighter binding to Sephadex. The majority of the fluorescently labelled DBD tetramers were retained on the Sephadex surface even after four months. Affinity columns generated using either approach effectively purified both SBP-tagged and biotinylated proteins. These columns are reusable and functional even after a year of frequent use.

## Introduction

Affinity chromatography is an efficient method allowing selective purification of target proteins from a crude extract in one step with a high degree of purity^1^. It is the method of choice for most protein purifications if available. The current approach to prepare the affinity matrices is relatively labor intensive and tedious. The capturing molecules for immobilization to the matrices first have to be highly purified and concentrated. They are then chemically coupled to the matrices. Since many residues (e.g. lysine) within a protein can be available for coupling, it is seldom possible to immobilize all these capturing molecules in an orientation specific manner^2,3^. Furthermore, some of the activated residues for coupling can be in or near the active site. The others when coupled to the matrix surface may block the accessibility of the interacting partners. All these factors can lower the binding capacity of the affinity matrices.

A recently reported molecular biological approach has greatly simplified this process^4^. Molecular fusion of the capturing molecule to an agarose-binding domain (ABD) combines the purification and coupling processes into one step. Loading of the recombinantly produced fusion proteins from the crude cell extract directly to agarose beads, followed by washing to remove the unbound molecules, enables the preparation of the affinity matrix in a fast, simple and efficient manner. Besides agarose, dextran-based matrices such as Sephadex, Superdex (dextran with cross-linked agarose) and Sephacryl (dextran cross-linked to bisacrylamide) are also widely used for bioaffinity chromatography^1,5^. To extend the fusion protein based bio-coupling approach for affinity matrix preparation, the feasibility to use a dextran-binding domain (DBD) as a matrix immobilization tool was explored. DBD used in this study is a well characterized domain^6^ from a monomeric *Leuconostoc mesenteroides* dextransucrase^7^. It has two key features. (1) It binds to dextran with high affinity (K_d_ ~2.9 nM). (2) This domain is relatively small. Most of the full-length dextran-binding domains in dextransucrases are 30–60 kDa in size^8^. Truncation of the 40-kDa C-terminal DBD to 14 kDa, while retaining the full binding strength as compared to the intact form, makes this domain ideal for fusion construction. The capturing molecule used in this study for preparing the affinity matrix is an engineered streptavidin (SAVSBPM18, abbreviated as M18). It has three attractive features^9^. (1) While retaining high affinity (K_d_ ~10^−8^ M) and specificity against both biotin and a short streptavidin-binding tag known as streptavidin-binding peptide (SBP) tag^10,11^, this designer streptavidin can bind both biotin and SBP in a reversible manner. In consequence, this streptavidin-based matrix can be applied to purify biotinylated or SBP-tagged proteins with high purity and recovery. (2) The affinity matrix can be easily regenerated under mild conditions and reused for many rounds. (3) Streptavidin (including M18) is a tetrameric protein with a biotin binding pocket in each subunit^12,13^. The tetrameric nature of the M18-DBD fusion can strengthen the retention of the fusions to Sephadex via the avidity effect.

This study used two approaches to generate the M18-DBD fusion. The first approach creates an SAVSBPM18-Linker-DBD (M18-L-DBD) fusion that allows the purification and immobilization of the fusion to Sephadex in one step. This represents the easiest way to prepare the affinity matrix. The second approach aims to develop a flexible means to prepare many different affinity matrices in a simple and efficient manner. This approach takes advantage of the efficient covalent assembly of a 12-amino-acid peptide tag known as SnoopTag (ST) with its 12-kDa specific interaction partner, SnoopCatcher (SC)^14^. This tag-catcher system was elegantly developed by splitting the D4 domain in the *Streptococcus pneumonia* adhesin (RrgA) into two components. Binding of SnoopTag to SnoopCatcher results in the formation of a covalent isopeptide bond between the tag and the catcher. A SnoopCatcher-Linker-DBD (SC-L-DBD) was constructed. This fusion can potentially capture any SnoopTagged proteins including M18-Linker-SnoopTag (M18-L-ST). Optimal conditions for generating Sephadex matrices with immobilized M18-L-ST·SC-L-DBD complexes were explored and established (· represents an isopeptide linkage).

Fluorescently labelled monomeric SC-L-DBD and tetrameric M18-L-ST·SC-L-DBD complexes confirmed that these fusions have the ability to bind to Sephadex G-100. Their retention on the bead surface with time reflects the binding strengths of these constructs and the suitability of DBD for the immobilization applications.

Matrices prepared from both the direct fusion and the M18-L-ST·SC-L-DBD complexes offered clean purification with high recovery of an SBP-tagged reporter and two biotinylated proteins (4.8 and 12 biotin moieties per protein). These columns are reusable and functional even after being packed for more than a year. Since dextran is commonly used in both the academic^15^ and industrial/medical sectors^16,17^, potential applications of the M18-DBD fusion and its derivatives will be discussed.

## Results and Discussion

### Features of M18-L-DBD and its recombinant production in *E. coli*

**Figure 1:**
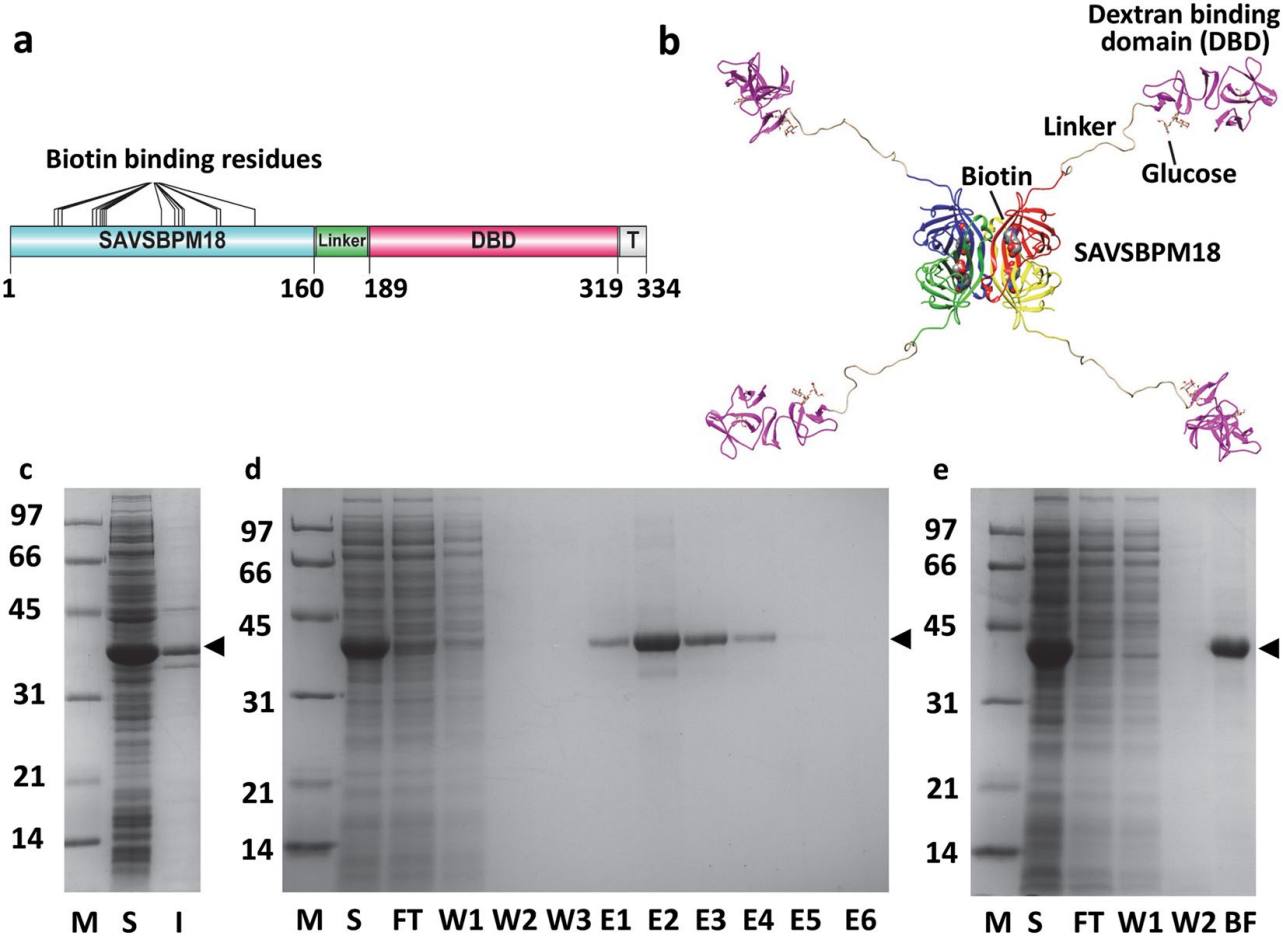
Primary and modelled structures of SAVSBPM18-Linker-Dextran-binding domain (M18-L-DBD), and its purification and immobilization to Sephadex. (**a**) Organization of various domains [engineered streptavidin SAVSBPM18, linker, dextran-binding domain (DBD) and a short C-terminal tail sequence (T)] in M18-L-DBD. Amino acid residues that mark the boundary of the domains are listed. (**b**) A modelled structure of M18-L-DBD. The four subunits in SAVSBPM18 are colored in red, yellow, green and blue, respectively. DBD is colored in purple. (**c**) SDS-PAGE showing intracellular production of M18-L-DBD in *E. coli* BL21[pET29B-M18-L-DBD]. (**d**) Purification of M18-L-DBD using biotin-agarose. Fractions were analyzed by SDS-PAGE. (**e**) SDS-PAGE analysis of the binding of M18-L-DBD to Sephadex G-100. All samples in panels c–e were boiled before loading. Arrowhead indicates the position of M18-L-DBD. M, molecular weight markers (sizes in kDa); S, intracellular soluble fraction; I, intracellular insoluble fraction; FT, flow-through fraction; W, wash fractions; E, elution fractions; BF, bound fraction. Gel profiles shown in panels c, d and e are from different gels.

The organization of the domains in M18-L-DBD is illustrated in Fig. 1a. A 28-amino-acid glycine rich sequence (Supplementary Sequence 1) serves as a flexible linker for two functions. (1) With the unstructured linker as a spacer, both the streptavidin subunit and the dextran-binding domain can fold independently without interference from each other. (2) Even if all four dextran-binding domains in each assembled tetrameric fusion anchor to a Sephadex bead, the streptavidin subunits can be projected away from the dextran-binding domains. Based on the three-dimensional structure of the modelled M18-L-DBD fusion (Fig. 1b), room should be available to make the biotin binding sites accessible for the binding of biotinylated or SBP tagged proteins. M18-L-DBD was produced predominantly in the intracellular soluble fraction of *E. coli* (Fig. 1c). The protein monomer migrated on an SDS-polyacrylamide gel with an apparent molecular mass around 38 kDa. This agrees closely with the expected value (35 kDa). The protein production yield was estimated to be exceeding 300 mg/l of cell culture.

### M18-L-DBD retains the biotin binding capability

M18-L-DBD should have both the reversible biotin-binding attribute and the dextran immobilization capability. To evaluate its biotin binding capability, M18-L-DBD was applied to a biotin agarose column. Figure 1d shows that M18-L-DBD was selectively bound to biotin agarose. Large amounts of pure protein could be easily recovered from the intracellular soluble fraction by affinity chromatography using biotin agarose and elution with biotin.

### Immobilization of M18-L-DBD to Sephadex G-100

To evaluate the dextran binding functionality and to prepare the M18-L-DBD affinity matrix, M18-L-DBD was applied to a Sephadex G-100 column using two strategies. In the first strategy M18-L-DBD affinity-purified by biotin agarose was loaded to the column. This worked well. However, the purified proteins in a highly concentrated state tend to form precipitates. This results in a significant loss of the starting material. An alternative and more simple approach is to load the crude intracellular soluble fraction containing M18-L-DBD directly to Sephadex-G100 (Fig. 1e). M18-L-DBD in the crude sample was applied under a slightly overloaded condition. Analysis of the boiled matrix (BF in Fig. 1e) showed that M18-L-DBD was selectively retained on the column with over 95% of the bound proteins composed of M18-L-DBD. Dialysis of the crude sample prior to column loading is not crucial. This simple method simultaneously affinity-purifies and immobilizes M18-L-DBD to generate the M18-L-DBD matrix in one step. By overloading the column with M18-L-DBD, around 708 ± 13 µg (5 nmoles of tetramers, Table 1) of M18-L-DBD could be captured per ml of Sephadex G-100.

**Table 1:** Binding of biotinylated BSA to M18-L-DBD matrix, M18-L-ST·SC-L-DBD matrix and M18-Affigel.

SAV	Matrix	Coupling method	SAV coupled (µg)	SAV coupled (nmole)	Biotin binding site (nmole)	BSA binding capacity (µg)	BSA binding capacity (nmole)	nmole BSA/ nmole SAV	Capture efficiency (%)
M18-L-DBD (Fully saturated matrix)	Sephadex	biocoupling	708 ± 13*	5	20	485 ± 3*	7.30	1.46	73.0
M18-L-DBD (Half saturated matrix)	Sephadex	biocoupling	354	2.5	10	252 ± 2.9*	3.79	1.52	75.8
M18-L-ST·SC-L-DBD (Fully saturated matrix)	Sephadex	biocoupling	610 ± 6*	2.85	11.4	NA	NA	NA	NA
M18-L-ST·SC-L-DBD (Half saturated matrix)	Sephadex	biocoupling	305	1.43	5.72	143 ± 2.0*	2.15	1.50	75.2
M18	Affi-gel	Chemical coupling	1,000	15.1	60.4	981 ± 5.0*	14.77	0.98	48.9
Recombinant SAV (Commercial)	Sepharose CL-6B	Chemical coupling	239–318**	4.5–6**	18–24	NA	NA	NA	NA

Table 1. 1-ml columns containing one of the affinity matrices were overloaded with biotinylated BSA. For each matrix, the amount in flow-through and wash fractions containing biotinylated BSA was quantitated by Bio-Rad Protein Assay Dye Reagent. The amount captured was estimated as the balance between the amount loaded and the amount in the flow through plus wash fractions. BSA used in this study has 12 biotin moieties per protein. Number of biotin binding site in each column is calculated by the number (nmoles) of M18 (and its derivatives) immobilized to the matrix × 4 since each streptavidin has four biotin binding sites. Capture efficiency is determined based on the assumption that one tetrameric streptavidin can bind two biotinylated BSA proteins. The molecular weight for the monomer of M18, M18-L-DBD, M18-L-ST·SC-L-DBD, recombinant streptavidin (commercial) and BSA is 16,519, 35,038, 53,426, 13,250 and 66,430, respectively. * indicates that the value represents an average of three trials. Data are expressed as average ± SD. ** indicates that the values were estimated based on the number of biotin binding site. NA: data not available.

### Recombinant production of SnoopCatcher-L-DBD (SC-L-DBD) and M18-L-SnoopTag (M18-L-ST)

The direct fusion approach requires M18 and DBD to be folded independently without interfering with each other and the fusion to be in the soluble state. As mentioned above, purified M18-L-DBD at high concentrations tends to form insoluble precipitates. It would be of interest to explore other options as a backup. One possibility is to first produce M18 and DBD individually in a soluble and functional state. These components can then be biologically ligated together via covalent bond formation to generate the final soluble fusion products.

**Figure 2:**
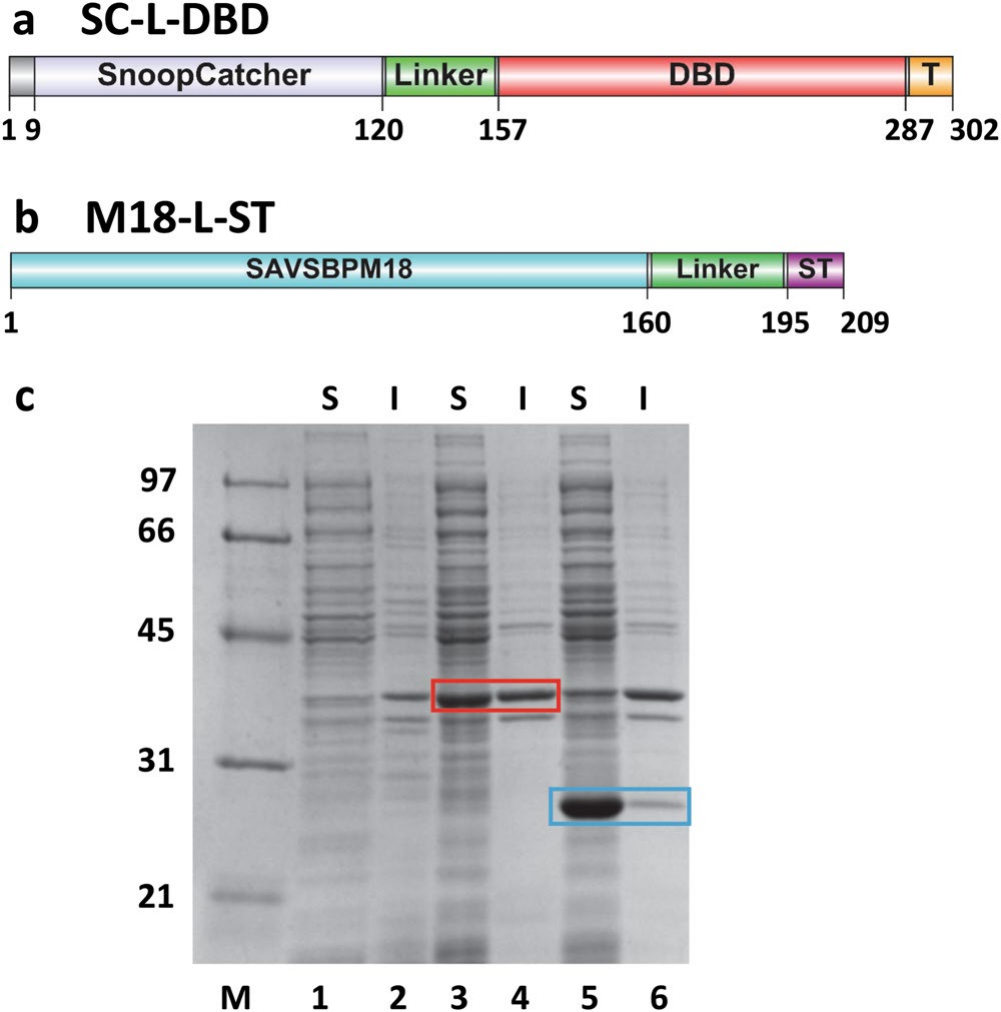
Domain organization and production of SnoopCatcher-Linker-dextran-binding domain (SC-L-DBD) and SAVSBPM18-Linker-SnoopTag (M18-L-ST). (**a**) A schematic drawing of various segments [a short N-terminal sequence, SnoopCatcher, linker, dextran-binding domain (DBD) and a short C-terminal tail sequence (T)] in SC-L-DBD. (**b**) A schematic drawing of various domains [SAVSBPM18, linker and SnoopTag (ST)] in M18-L-ST. Amino acid residues that mark the boundary of the key domains in panels a and b are listed. (**c**) Intracellular production of SC-L-DBD from *E. coli* BL21[pET29B-SC-L-DBD] and M18-L-ST from *E. coli* BL21[pET-29B-M18-L-ST]. Samples were analyzed by SDS-PAGE and boiled before loading to the gel. Lanes 1, 2 are the intracellular fractions from *E. coli* BL21 [pET29B]. These samples serve as the negative control. Lanes 3, 4 are the intracellular fractions from *E. coli* BL21 [pET29B-SC-L-DBD]. SC-L-DBD is boxed in red. *E. coli* BL21[pET29B] has an endogenous protein which comigrates at the same position as SC-L-DBD. Lanes 5, 6 are the intracellular fractions from *E. coli* BL21 [pET29B-M18-L-ST]. M18-L-ST is boxed in blue. M, molecular weight markers (sizes in kDa); S, soluble fraction; I, insoluble fraction.

An approach using two heterodimeric coiled coil sequences^18,19^ to link M18 to DBD has been evaluated. However, the heterodimerization efficiency was low. We here explored another alternative using the SnoopCatcher (SC)-SnoopTag (ST) based bio-ligation system^14^. Both SC-L-DBD (Fig. 2a, Supplementary Sequence 2) and M18-L-ST (Fig. 2b, Supplementary Sequence 3) were created. This approach offers great flexibility to join any SnoopTagged proteins to SC-L-DBD. Large amounts of SC-L-DBD and M18-L-ST were produced in the intracellular soluble fractions of *E. coli* (Fig. 2c). The production yield of soluble proteins is around 70 mg/l for SC-L-DBD and 140 mg/l for M18-L-ST, respectively. SC-L-DBD (expected MW 32,415) migrated on an SDS-polyacrylamide gel with an apparent molecular mass of 35 kDa while the monomer of M18-L-ST (expected MW 21,011) migrated with an apparent molecular mass of 27 kD. Presence of a flexible linker in a fusion protein usually results in a slight increase in the apparent molecular mass of the fusion^20^.

### Approaches to generate Sephadex bound M18-L-ST·SC-L-DBD

**Figure 3:**
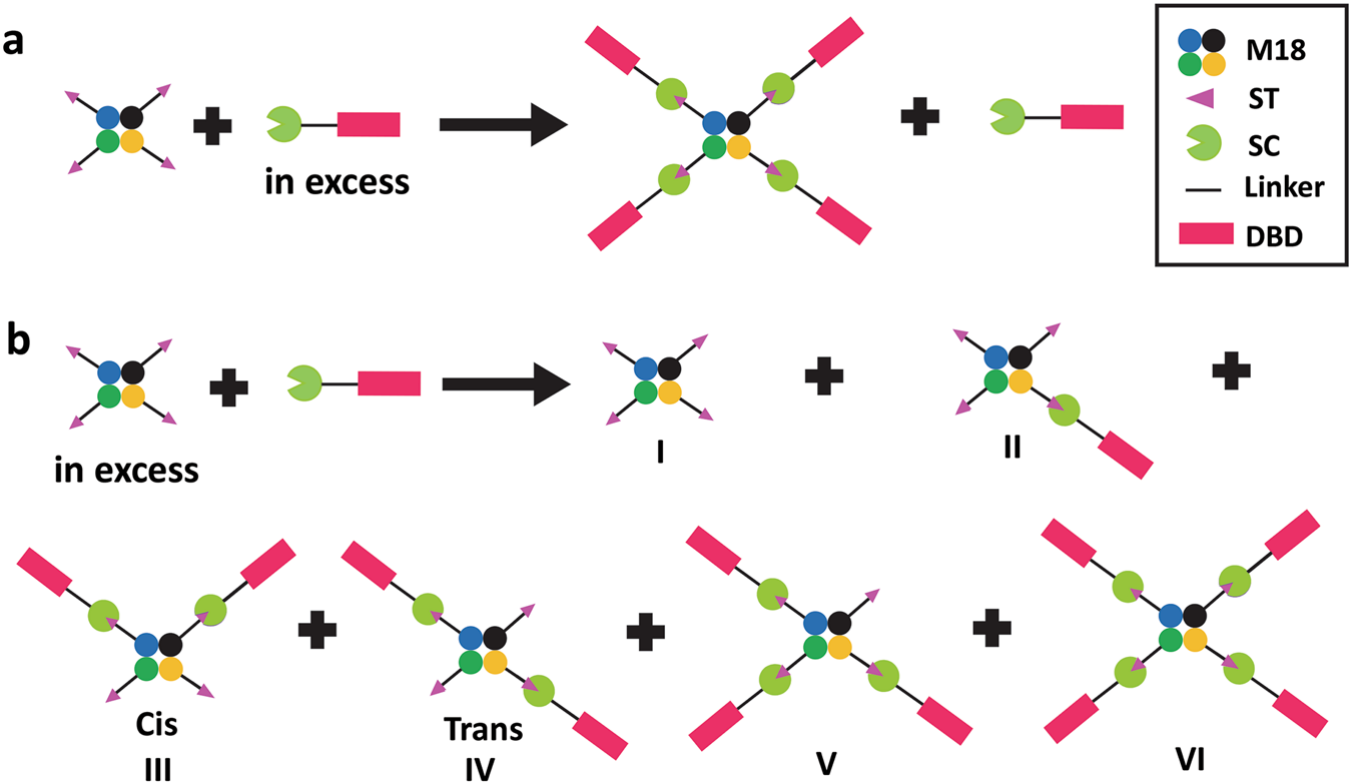
Two approaches to generate M18-L-ST·SC-L-DBD for affinity matrix preparation. Generation of the covalently linked M18-L-ST·SC-L-DBD complexes by mixing (**a**) M18-L-ST with excess SC-L-DBD or (**b**) SC-L-DBD with excess M18-L-ST. M18, SAVSBPM18; ST, SnoopTag; SC, SnoopCatcher; L, Linker; DBD, Dextran-binding domain.

The initial strategy of producing Sephadex bound M18-L-ST·SC-L-DBD is to first couple SC-L-DBD onto a Sephadex G-100 column and then to saturate the immobilized SC-L-DBD with M18-L-ST. The covalent complex M18-L-ST·SC-L-DBD was obtained but with low efficiency. Even with overnight incubation involving different loading amounts of M18-L-ST, large amounts of free SC-L-DBD and M18-L-ST remained unreacting. Mixing SC-L-DBD bound Sephadex beads in a batch mode (instead of a column format) with excess M18-L-ST did not improve the coupling reaction. The limited diffusion rate of the Sephadex bound SC-L-DBD can be a major factor contributing to the observed inefficient coupling. To address this concern, production of the covalent complex in solution first has to be optimized. The complexes will then be purified and immobilized to Sephadex. To generate the M18-L-ST·SC-L-DBD complexes, the bio-conjugation process could be performed with either SC-L-DBD or M18-L-ST in excess. Generation of M18-L-ST·SC-L-DBD under the condition with excess SC-L-DBD is the preferred choice (Fig. 3a). It can easily generate a homogenous population of M18-L-ST·SC-L-DBD with four SC-L-DBD modules covalently linked to each M18-L-ST tetramer. In contrast, the other condition would generate a heterogeneous population M18-L-ST·SC-L-DBD with one to four SC-L-DBD modules per M18-L-ST tetramer (Fig. 3b). Relative to M18-L-ST·SC-L-DBD with four covalently linked SC-L-DBD modules, M18-L-ST·SC-L-DBD with less than four SC-L-DBD modules per M18-L-ST tetramer would have lower binding affinity towards Sephadex. Separation of the high affinity version from other lower affinity versions requires extra purification steps. Furthermore, the yield of the high affinity version would be lower than that generated by using excess SC-L-DBD.

### Generation of the M18-L-ST·SC-L-DBD matrix: SC-L-DBD in excess in the reaction mixture

The average production yield of the soluble SC-L-DBD (2.2 nmoles/ml of culture) is about a third of the soluble M18-L-ST (6.6 nmoles of monomer/ml of culture). SC-L-DBD has to be mixed with M18-L-ST at a ratio greater than 3:1 (in terms of culture volume) so that SC-L-DBD will be in excess. Under this condition, M18-L-ST·SC-L-DBD and SC-L-DBD will be the major species in the reaction mix after completion of the bio-conjugation reaction (Fig. 3a).

**Figure 4:**
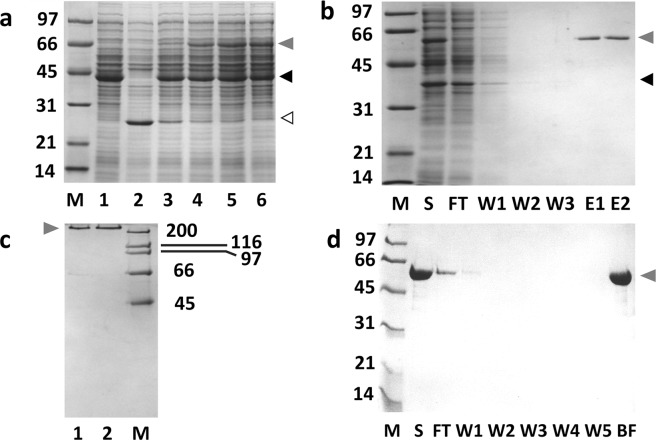
Formation of M18-L-ST·SC-L-DBD with excess SC-L-DBD in the reaction mix and the preparation of the M18-L-ST·SC-L-DBD matrix (**a**) Kinetics of M18-L-ST·SC-L-DBD formation. Samples were analyzed by SDS-PAGE. Lane 1 is the soluble fraction containing SC-L-DBD. Lane 2 is the soluble fraction containing M18-L-ST. Lanes 3–6 represent the reaction mixtures that were post-mixed for 5 minutes, 90 minutes, 3 hours and overnight, respectively. (**b**) Biotin agarose column chromatography of the reaction mix. (**c**) SDS-PAGE analysis of the tetrameric state of M18-L-ST·SC-L-DBD complexes. Elution fractions (E1 and E2 in panel b) from the biotin agarose column were pooled and dialyzed against PBS. Lanes 1 and 2 show the purified M18-L-ST·SC-L-DBD complexes in the absence and presence of biotin, respectively. Samples in this panel (except the high molecular weight markers) were not boiled. (**d**) Binding of the elution fractions from biotin agarose column (panel b) to Sephadex G-100. Elution fractions were pooled, concentrated, dialyzed against PBS and applied to a column of Sephadex G-100. Fractions were analyzed by SDS-PAGE. Samples in panels a, b and d were boiled before gel loading. M, molecular weight markers (sizes in kDa); S, sample; FT, flow-through fraction; W, wash fractions; (**E**) elution fractions; BF, bound fraction. Open arrowhead, M18-L-ST; Black closed arrowhead, SC-L-DBD; Grey closed arrowhead, M18-L-ST·SC-L-DBD. Gel profiles shown in panels a–d are from different gels.

Figure 4a shows the kinetics of the M18-L-ST·SC-L-DBD formation at 4 °C. In this reaction, SC-L-DBD was mixed with M18-L-ST at a ratio of 6:1 (in terms of culture volume). M18-L-ST was gradually depleted and a new covalent complex, M18-L-ST·SC-L-DBD, was formed (Fig. 4a, lanes 3–6). This complex migrated on the SDS-polyacrylamide gel with an apparent molecular mass of 66 kDa, which agrees closely with the expected apparent molecular mass of 62 kDa (35 kDa + 27 kDa). Some covalent complexes could be observed just five minutes post-mixing. Formation of covalent complex was more than 70% complete at 90 min and over 90% complete at 3 hours post-mixing. Overnight reactions are usually carried out to ensure completion of the reaction.

M18-L-ST·SC-L-DBD could be separated from the excess SC-L-DBD and other background proteins by affinity chromatography using biotin agarose (Fig. 4b). While all the free SC-L-DBD molecules and background contaminants were out in the flow-through and wash fractions, highly pure M18-L-ST·SC-L-DBD was captured on the column and could be recovered in the elution fractions (Fig. 4b, E1 and E2) using biotin as the eluent. The absence of M18-L-ST in the elution fractions indicates that all M18-L-ST moieties have reacted to form the covalent complexes. When the purified M18-L-ST·SC-L-DBD complexes were not boiled before loading onto the polyacrylamide gel, these complexes migrated with an apparent molecular mass over 200 kDa (Fig. 4c, lanes 1 and 2) which corresponds to the expected apparent molecular mass of 264 kDa (66 kDa × 4) of the assembled tetramer. This assembly process is relatively biotin independent (Fig. 4c, lane 1 vs lane 2). The faint band observed in lane 1 represents small quantities of disassembled M18-L-ST·SC-L-DBD monomers. The partial disassembly of tetrameric M18-L-ST·SC-L-DBD in the absence of biotin can be triggered by the presence of SDS. In contrast, biotin binding is known to strengthen the subunit interactions and can minimize the disassembly of tetrameric streptavidin even in the presence of SDS^21^ (Fig. 4c, lane 2).

Purified M18-L-ST·SC-L-DBD could be effectively captured by Sephadex G-100 to generate the M18-L-ST·SC-L-DBD matrix (Fig. 4d). Analysis of the bound fraction (BF in Fig. 4d) by boiling a bead sample suggests that the covalent complex captured was composed purely of M18-L-ST·SC-L-DBD. By overloading the column with M18-L-ST·SC-L-DBD, the amount of the immobilized M18-L-ST·SC-L-DBD was estimated to be around 610 ± 6 µg (2.75 nmoles of tetramers, Table 1) per ml of Sephadex G-100.

### Dynamic spatiotemporal distribution of fluorescently labelled His-SC-L-DBD(Cys) and M18-L-ST·His-SC-L-DBD(Cys) to Sephadex G-100 beads

**Figure 5:**
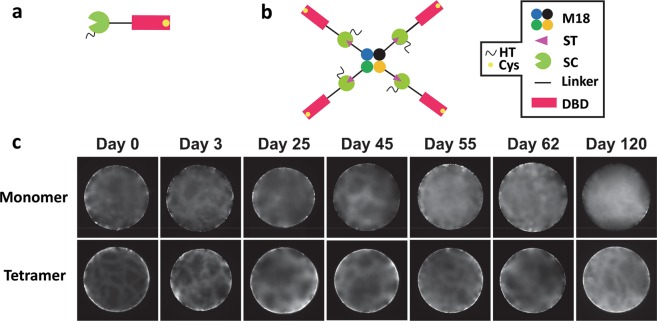
Binding of the fluorescently labelled monomeric His-SC-L-DBD(Cys) and tetrameric M18-L-ST·His-SC-L-DBD(Cys) to Sephadex G-100 beads. (**a**,**b**) Domain organization of His-SC-L-DBD(Cys) and M18-L-ST·His-SC-L-DBD(Cys). HT, His-tag; Cys, cysteine; M18, SAVSBPM18; ST, SnoopTag; SC, SnoopCatcher; DBD, Dextran-binding domain. (**c**) Spatial and temporal distribution of fluorescently labelled monomeric His-SC-L-DBD(Cys) and tetrameric M18-L-ST·His-SC-L-DBD(Cys) to Sephadex G-100 beads. Labelled protein binding to Sephadex was analyzed up to 120 days after mixing the labelled proteins with the beads. Surface distribution of the fluorescently labelled molecules is the major focus of these pictures.

In this study, DBD is applied as an immobilization domain to anchor capturing proteins to chromatographic matrices such as Sephadex to generate the affinity matrices. It is interesting to monitor the retention ability of the bound DBD fusions in their monomeric and tetrameric states to Sephadex using fluorescent microscopy^4,22^. A His-Tagged version of SC-L-DBD with a cysteine in the C-terminal region was constructed (Fig. 5a, Supplementary Sequence 4). The His-Tag enables purification of His-SC-L-DBD(Cys) by IMAC affinity chromatography^23^. Presence of a unique cysteine allows the fluorescent labelling of this protein through thiol coupling^4^. Pure M18-L-ST·His-SC-L-DBD(Cys) complexes with four DBD domains per tetrameric M18 (Fig. 5b) were generated by the approach using excess His-SC-L-DBD(Cys). The use of the direct fusion version of M18-L-DBD(Cys) for the fluorescent study is not possible because this fusion was found to become insoluble after fluorescent labelling. DBD fusions in either the monomeric or tetrameric state are mainly localized on the Sephadex bead surface from day 0 to day 62 (Fig. 5c). Events of dissociation, diffusion and rebinding of DBD to dextran strands result in the diffusion of the molecules into the centre of the beads. This effect is much more dramatic with monomeric DBD. The disappearance of most of the surface bound DBD moieties and the increase in brightness at the centre of the bead at day 120 were obvious. In contrast, the majority of tetrameric DBD complexes are retained on the bead surface even after four months. This illustrates that avidity is an effective means to strengthen the retention of tetrameric DBD on the Sephadex surface.

Since the binding affinity of DBD to dextran is in the nmolar range^6^ while the binding strength of agarose-binding domain to agarose is in the µmolar range^4,22^, the retention ability of the bound monomeric or tetrameric DBD to the Sephadex surface is much better than that of the monomeric or tetrameric agarose-binding domain to agarose. This makes DBD an attractive immobilization tool for affinity matrix preparation.

The fluorescence intensity on the Sephadex bead surface is uneven (Fig. 5c). This is very different from the relatively even distribution of the bound agarose-binding domains on the agarose bead surface^4,22^. Scanning electron microscopic studies illustrate that agarose beads^24–26^ have a relatively smooth surface. In contrast, Sephadex beads have a crinkled surface^24,25^. Presence of an uneven bead surface can explain the observed uneven distribution of the surface bound fluorescently labelled DBD molecules.

### Application of M18-L-DBD matrix and M18-L-ST·SC-L-DBD matrix for affinity purification of proteins

The matrix half-saturated with immobilized M18-L-DBD (2.5 nmoles of M18-L-DBD per ml of Sephadex, Table 1) or M18-L-ST·SC-L-DBD (1.43 nmoles per ml of Sephadex) was applied to purify SBP-tagged β-lactamase^9,11^ and several biotinylated model proteins with different degrees of biotinylation. The rationale for the use of the half-saturated matrices is to ensure that any M18-DBD or M18-L-ST·SC-L-DBD detached from the matrix can easily rebind to the matrix again.

**Figure 6:**
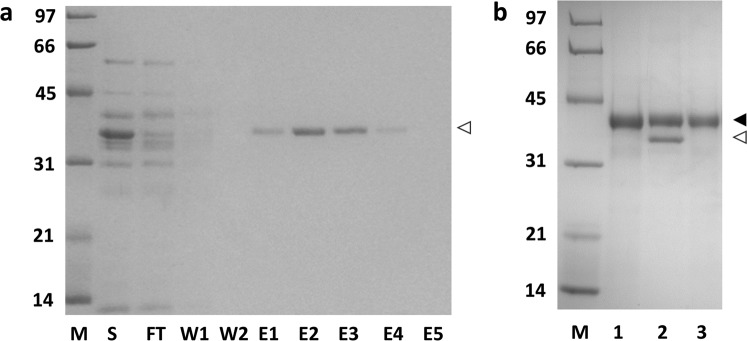
Purification of SBP-tagged β-lactamase using M18-L-DBD affinity matrix under a non-overloading condition. (**a**) Analysis of different fractions from the column by SDS-PAGE. S, *B. subtilis* culture supernatant containing overproduced SBP-tagged β-lactamase; FT, flow-through fraction; W, wash fractions; E, elution fractions. Open arrowhead indicates SBP-tagged β-lactamase. (**b**) SDS-PAGE of the bound fraction from the M18-L-DBD Sephadex column used for purification of SBP-tagged β-lactamase. Lane 1 is the boiled matrix before sample loading; lane 2 represents the boiled matrix after sample loading and before elution with biotin; lane 3 is the boiled matrix after elution with biotin. Closed arrowhead, M18-L-DBD; Open arrowhead, retained SBP-tagged β-lactamase. M, molecular weight markers (sizes in kDa). Gel profiles shown in panels a and b are from different gels.

For purification of SBP-tagged β-lactamase, the sample was applied to the affinity column under a non-overloading condition (i.e. not to exceed the column binding capacity of SBP-tagged protein)^9^. SDS-PAGE (Fig. 6 and Supplementary Fig. S1a) shows that the SBP-tagged protein could be selectively captured and affinity-purified in one step on either column. Recovery in both cases was around 80% within a few column volumes of elution. Both columns were functional even after one year of frequent use.

**Figure 7:**
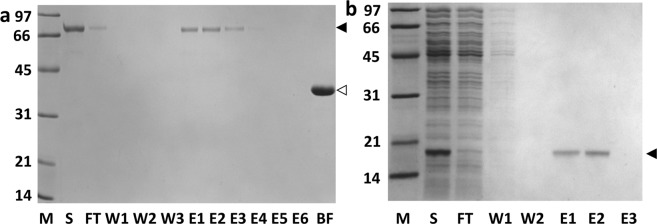
Affinity chromatography of biotinylated proteins using M18-L-DBD matrix. (**a**) Biotinylated bovine serum albumin (BSA) with 12 biotin moieties per molecule. Overloading amount of biotinylated BSA was applied. Fractions were analyzed by SDS-PAGE. Closed arrowhead, biotinylated BSA; Open arrowhead, Sephadex bound M18-L-DBD. (**b**) Biotinylated human fibroblast growth factor (FGF) with 4.8 biotin moieties per protein. Pure chemically biotinylated human FGF was mixed with the *E. coli* soluble fraction to constitute the crude sample. Fractions were analyzed by SDS-PAGE. Closed arrowhead, biotinylated FGF. M, molecular weight markers (sizes in kDa); S, loaded sample; FT, flow-through fraction; W, wash fractions; E, elution fractions; BF, bound fraction. Gel profiles shown in panels a and b are from different gels.

For capture of biotinylated proteins, chemically biotinylated bovine serum albumin (BSA) with an average of 12 biotin moieties per protein was first selected for testing with both matrices. BSA should bind tightly to the affinity matrix because of the avidity effect (i.e. one BSA can potentially interact with multiple M18). As expected, BSA binds well to both matrices (Fig. 7a for the M18-L-DBD matrix and Supplementary Fig. S1b for the M18-L-ST·SC-L-DBD matrix). To ensure that the bound protein could be efficiently eluted off the column, 10 mM (instead of 5 mM) of biotin was used as the eluent and the column was incubated with the eluent for 1–2 hours before collecting the eluted fractions. BSA could be eluted from either matrix with no difficulties (Fig. 7a and Supplementary Fig. S1b). To confirm that all bound BSA could be eluted from the column, a sample of the affinity matrix post-elution was boiled to analyze the bound fraction. As shown in Fig. 7a (lane BF) only M18-L-DBD was detected. The same is true for the M18-L-ST·SC-L-DBD matrix (lane BF of Supplementary Fig. S1b).

To estimate the binding capacity of both matrices, biotinylated BSA was applied at an overloading amount. Analysis of the protein distribution in the flow-through, wash and eluted fractions showed that at saturation, 1 ml of M18-L-DBD affinity matrix (containing 2.5 nmoles of tetrameric M18-L-DBD) could bind around 252 µg ± 3 µg (3.8 nmoles, Table 1) of biotinylated BSA. In a tetrameric streptavidin, two biotin-binding sites are localized on one side while the other two are on the opposite side (Fig. 1b and Supplementary Fig. S2)^13,27^. Since BSA is relatively bulky with a molecular mass of 66.43 kDa, binding of one BSA molecule to a biotin-binding pocket will likely hinder the binding of another BSA to the biotin-binding pocket on the same side (Supplementary Fig. S2). Therefore, one tetrameric streptavidin will likely bind a maximum of two BSA molecules, one on each side of the tetrameric streptavidin. Capture of a maximum of 5 nmoles of biotinylated BSA/ml matrix would be expected in this case. As one biotinylated BSA (12 biotin moieties/BSA) can interact with two or more tetrameric streptavidin, capture of 3.8 nmoles of BSA per ml matrix seems to be reasonable. This translates to a capture efficiency of 76% (Table 1). Similar binding efficiency was observed with the M18-L-ST·SC-L-DBD affinity matrix. In another study, the Sephadex matrix was fully saturated with M18-L-DBD (i.e. 5 nmoles of M18-L-DBD immobilized per ml of Sephadex). This matrix was found to capture biotinylated BSA with a capture efficiency of 73% (Table 1).

The second chemically biotinylated protein studied is the human fibroblast growth factor (FGF) with a biotin to protein ratio of 4.8. In comparison with BSA, this protein should have less avidity effect in binding. To demonstrate the selective purification of biotinylated FGF from a mixture of proteins, pure biotinylated FGF was mixed with a crude *E. coli* cell extract and the sample was loaded onto either column. Figure 7b and Supplementary Fig. S1c show that despite the lower avidity effect, FGF was effectively captured and selectively purified in one step by either matrix. No leakage of the protein was observed in the wash fractions. Protein recovery was in the range of at least 80% within a few column volumes.

Finally, two enzymatically biotinylated proteins [pure maltose binding protein (MBP-AviTag from Avidity) and a pure staphylokinase with biotinylation tag^28^] with one biotin per protein were used as the model proteins for purification. The binding of these proteins to either column was not tight enough to allow effective purification. The inability of these matrices to bind proteins with single biotin per protein is unexpected since the M18 affinity matrix (M18 Affi-gel) generated by chemical coupling has been successfully applied to purify monobiotinylated MBP-AviTag^9^. Replacement of M18 in M18-L-DBD with either another mutein that has a higher (yet still reversible) biotin binding affinity or a different linker may address this limitation. Should purification of monobiotinylated proteins be the major application, the use of M18 Affi-gel matrix^9^ is recommended.

A remarkable feature for the M18-DBD matrices is that the affinity capture, elution and regeneration processes can be operated under mild, protein-friendly conditions (i.e. PBS ± biotin). SBP tagged or biotinylated proteins are selectively and highly purified (Figs 6–7 and Supplementary Fig. S1). If more stringent conditions are needed, PBS or Tris-buffered saline containing mild detergents (Tween 20 or Triton X-100 to 0.1%) with different salt concentrations (0.1 M to 0.25 M NaCl) and different pH (7.0 to 8.0) have been tested. The experiments worked well under room temperature or at 4 °C. All these operating conditions do not trigger any detachment of the bound M18-DBD from the matrix.

### Comparison of the streptavidin affinity matrices generated by different coupling methods

Affinity matrices generated by bio-coupling (using the direct fusion and bio-ligation approaches) and chemical coupling were compared in three aspects. The first aspect is the coupling capacity of streptavidin per ml of settled beads. For the bio-coupling approach, each streptavidin subunit is connected to DBD via a linker sequence (Fig. 1b). A tetrameric streptavidin-DBD fusion can occupy a larger surface area of a Sephadex bead during the binding event. Under a fully saturated condition, 5 nmoles of tetrameric M18-L-DBD can be coupled to 1 ml of Sephadex (Table 1). With the presence of SnoopCatcher/SnoopTag in the linker region as the connection domains, it is not surprising that 1 ml of Sephadex can capture only 2.85 nmoles of M18-L-ST·SC-L-DBD. Chemical coupling of M18 to affi-gel offers a higher coupling capacity and can reach over 15 nmoles per ml of gel. In comparison, a commercially available streptavidin-agarose was reported to have 18–24 nmoles of biotin binding sites per ml of matrix. Therefore, the number of biotin binding sites (20 nmoles) available from 1 ml of the M18-L-DBD Sephadex is fairly comparable.

The second aspect is the capture efficiency of the matrices. With the ability to chemically couple more M18 to affi-gel, this matrix can capture more biotinylated BSA (Table 1). However, on molar basis, one mole of chemically coupled tetrameric M18 could only capture 0.98 mole of biotinylated BSA (Table 1). In contrast, one mole of tetrameric M18-L-DBD generated by bio-coupling could capture approximately 1.46 moles of biotinylated BSA. Thus, the effective column binding capacity (on molar basis) is considerably lower with the matrix generated by chemical coupling. Assuming one tetrameric streptavidin can bind two biotinylated BSA molecules with one BSA on either side of the tetrameric streptavidin (Supplementary Fig. 2), the capture efficiency (Table 1) of biotinylated BSA by M18-affi-gel is only 48.9% while the capture efficiency for columns generated by bio-coupling can reach 73–76%. Among other factors, the lack of orientation-specific capturing molecules and the indiscriminating activation of lysine residues (some of which might be critical for the capturing function) of M18 during chemical coupling might be contributing to this lower effective binding capacity of the M18 Affi-gel.

The third aspect is the simplicity of matrix preparation. The chemical coupling approach is the most tedious and labor intensive process, requiring three steps including purification, concentration of the capturing agent (e.g. M18), and coupling to the activated matrix. The coupling efficiency is also more difficult to be precisely controlled. In contrast, the direct fusion approach of bio-coupling is the simplest since purification, concentration and immobilization of M18-L-DBD can be consolidated into one-step. For the M18-L-ST·SC-L-DBD approach, although the use of the biotin agarose chromatography is required to remove the excess SC-L-DBD before loading onto the Sephadex, it is not more tedious than the chemical coupling approach. Furthermore, it does not require the use of the expensive, chemically activated matrix for coupling. It is important to note that the extra operation steps evoked in the M18-L-ST·SC-L-DBD system are peculiar in this case because it involved a tetrameric protein. For other monomeric proteins (e.g. monomeric streptavidin), the generation of affinity matrices by this SnoopCatcher-SnoopTag system can be as straightforward and efficient as the direct fusion approach. Crude extracts containing SC-L-DBD and excess SnoopTagged protein can be mixed in a proper ratio for several hours. The bio-ligated sample can then be applied directly to Sephadex to generate the affinity column. This system offers a high degree of flexibility since any SnoopTagged proteins can bio-ligate to SC-L-DBD for affinity matrix preparation.

### Potential applications

Although this study focuses on the simplified preparation of the M18-Sephadex matrix, many other dextran-based matrices including Sephacryl, Superdex and dextran coated silica monolithic media^28^ can serve as the support matrices.

Since a wide variety of engineered streptavidin^13,29,30^ is available with the biotin binding affinity ranged from lower to higher than wild-type streptavidin, both the wild-type streptavidin and the engineered versions such as streptactin^31^, SAVSBPM32^30^ and traptavidin^29^ can be fused to DBD. The resulting fusions can be applied for affinity purification and reversible/irreversible immobilization of target proteins. With the SnoopCatcher-L-DBD module described in this study, any proteins of interest fused to a SnoopTag can be easily coupled to the dextran matrices. Furthermore, technologies for preparing dextran-coated biosensor chips^32^, quantum dots^33,34^, magnetic nanoparticles^35,36^ and biodegradable hydrogels^17,37^ are well established. Coupling of fusions of engineered streptavidin and other proteins of interest to these platforms can be applied for biomolecular interaction studies, single-cell imaging, affinity protein purification, cell isolation, tumor targeting, tissue repair and drug delivery.

Two other potential applications of this system are worth attention. The first is for the easy isolation of genetically engineered therapeutic T cells for immunotherapy. Based on the chimeric antigen receptor (CAR)-T cell technology^38,39^, an antigen specific binding domain (e.g. a tumor specific single-chain antibody fragment) is fused to the transmembrane domain and the intracellular signalling domain of a T cell receptor to create a chimeric antigen receptor. The gene of this rationally designed receptor is then transduced to the T cells isolated from the patient using a viral vector. Reintroduction of these engineered cells to the patient usually shows promising efficacy for the treatment of a specific disease. Since the transduction efficiency is not 100%, a mixture of transduced and non-transduced T cells will be introduced to the patient. This creates variation in efficacy for the treatment. To address this concern, an affinity tag has been introduced to CAR^40^. Transduced cells can then be isolated using the affinity beads coated with the tag specific capturing agents. Based on the same principle, an SBP tag can be introduced to CARs. The transduced cells can be selectively isolated using dextran beads coupled with M18-L-DBD. Enriched cells are then introduced to the patient.

The second application is the easy generation of artificial antigen presenting cells to stimulate specific T cells^41^. This can be easily achieved by coupling both the biotinylated MHC-peptide complexes and co-stimulatory proteins to the dextran-coated nanoparticles displaying streptavidin-DBD fusions. The resulting nanoparticles can subsequently serve as artificial antigen presenting cells.

Essentially, many other potential applications of the M18-DBD system are possible and can be tailored depending on the needs of the users.

## Methods

### Plasmid construction

All synthetic genes were obtained from Bio Basic Canada. The synthetic gene encoding SAVSBPM18-linker-DBD (M18-L-DBD, Supplementary Sequence 1) was released from pUC57-M18-L-DBD through the NdeI and EcoRV digestion. The purified gene fragment was then inserted to NdeI/EcoRV cut pET29B to generate pET29B-M18-L-DBD.

To generate the SnoopCatcher-Linker-DBD (SC-L-DBD, Supplementary Sequence 2) expression vector, the synthetic gene encoding a 380-bp SC-L-DBD fragment was digested from pUC57-SC-L-DBD with NdeI and SexAI and inserted to NdeI/SexAI digested pET29B-M18-L-DBD.

The synthetic gene encoding SAVSBPM18-Linker-SnoopTag (M18-L-ST, Supplementary Sequence 3) was digested with NdeI and BlpI and inserted to NdeI/BlpI cut pET29B to generate pET29B-M18-L-ST. To construct the expression vector (pET29B-SC-L-DBDC) to produce SC-L-DBD(Cys) for fluorescent study, the synthetic gene encoding SC-L-DBD(Cys) was digested with AflII and NotI and inserted to AflII/NotI cut pET29B-SC-L-DBD. To generate the expression vector (pET19B-His-SC-L-DBDC) to produce the HisTag version of SC-L-DBD(Cys) (Supplementary Sequence 4), pET29B-SC-L-DBDC was digested by NdeI and BlpI and inserted to NdeI/BlpI cut pET19B which has a specific sequence encoding an N-terminal His tag.

The plasmids were transformed into *E. coli* BL21(DE3) which serves as the expression host.

### Cell culture and extraction of intracellular fractions for protein studies

Bacteria with pET29B-based plasmid were cultured in a shake flask at 30 °C in LB broth containing kanamycin (30 mg/l) until absorbance at 600 nm reached 0.8–1.0. IPTG was added to a final concentration of 0.2 mM to induce the expression of the inserted gene. Cell growth continued at 26 °C for 3–4 hours. Cells were harvested by centrifugation. Ampicillin (75 mg/l) was used to culture bacteria with pET19B-based plasmid.

For all extraction of intracellular proteins except where indicated, cell pellets were resuspended in PBS buffer (sodium phosphate 50 mM, NaCl 150 mM, pH 7.2) and disrupted by a French pressure cell press (Spectronic Instruments). The soluble fraction was separated from the insoluble fraction by centrifugation (17,000 g, 4 °C, 20 min).

### Affinity chromatography using biotin agarose

0.8 ml matrix of biotin agarose (Sigma) was used in column chromatography. Equilibration and wash buffers were PBS. Elution buffer was PBS containing 5 mM biotin.

### Immobilization of M18-L-DBD to Sephadex G-100

Soluble fraction containing M18-L-DBD (2–3 ml crude sample) was loaded to a 1-ml column of Sephadex G100 (GE Healthcare life Sciences). Contact time of sample with the column was allowed for an hour at room temperature before washing the column with PBS.

### Optimized conditions for the generation of M18-L-ST·SC-L-DBD with SC-L-DBD in excess in the reaction mixture

Immediately after cell disruption and removal of the intracellular insoluble fraction, the soluble fraction containing SC-L-DBD was mixed with the soluble fraction containing M18-L-ST at a ratio of 6:1 (in terms of the original culture volume). Alternatively (and for greater convenience), one can achieve the same result by mixing cell pellets harvested from the 2 cultures using a ratio of 6:1 (original cell culture volumes for SC-L-DBD: M18-L-ST) prior to cell disruption. The soluble fraction containing both SC-L-DBD and M18-L-ST was dialyzed against PBS at 4 °C overnight to allow thorough formation of M18-L-ST·SC-L-DBD.

### **P**urification of His-SC-L-DBD(Cys) and M18-L-ST·His-SC-L-DBD(Cys) for fluorescent microscopy

His-SC-L-DBD(Cys) in the soluble fraction of *E. coli* BL21(DE3)[pET19B-His-SC-L-DBDC] was purified by IMAC affinity chromatography. Cell pellet was disrupted in equilibration buffer (sodium phosphate 50 mM, NaCl 300 mM, imidazole 15 mM, pH 8.0). The soluble fraction was loaded to a column of His-Select Nickel Affinity gel (Sigma) and washed with the equilibration buffer. His-SC-L-DBD(Cys) was eluted by elution buffer (sodium phosphate 50 mM, NaCl 300 mM, imidazole 300 mM, pH 8.0). Elution fractions containing the pure proteins were pooled, concentrated and dialyzed against PBS. Protein concentration was determined using Bio-Rad Protein Assay Dye Reagent.

To generate M18-L-ST·His-SC-L-DBD(Cys), cell pellet from 50 ml culture of *E. coli* BL21(DE3) [pET19B-His-SC-L-DBD(Cys)] was resuspended in PBS with the pellet from 10 ml culture of *E. coli* BL21[pET29B-M18-L-ST] before cell disruption. The intracellular soluble fraction was gently rocked overnight at 4 °C to allow formation of the covalent complex M18-L-ST·His-SC-L-DBD(Cys) with depletion of all M18-L-ST. M18-L-ST·His-SC-L-DBD(Cys) was affinity-purified by biotin agarose (Sigma) column chromatography. After extensively washing the column with PBS, the bound protein was eluted by PBS containing 5 mM biotin. Fractions of interest were pooled, concentrated and dialyzed against PBS. Protein concentration was determined using Bio-Rad Protein Assay Dye Reagent.

To prepare dye conjugates of His-SC-L-DBD(Cys) and M18-L-ST·His-SC-L-DBD(Cys) for fluorescence microscopy, Alexa Fluor 594 C5 maleimide (Molecular Probes) was conjugated to the purified proteins according to the manufacturer’s instruction. After reaction, excess free dyes were removed by extensive dialysis against PBS.

### Spatiotemporal studies of the fluorescently labelled monomeric His-SC-L-DBD(Cys) and tetrameric M18-L-ST·His-SC-L-DBD(Cys) on Sephadex G-100 by fluorescence microscopy

A 100-μl Sephadex G-100 slurry (in a 50% suspension format) was mixed with 500 μl labelled proteins [containing 1 mg of Alexa Fluor 594 labelled monomeric His-SC-L-DBD(Cys) or tetrameric M18-L-ST·His-SC-L-DBD(Cys)]. The sample (in a total volume of 600 μl) was rotated by using an end-over-end rotator for 15 minutes at room temperature. The beads were then washed 5 times with 1 ml Tris-Buffered Saline (TBS: 50 mM Tris-HCl, pH 7.5; 150 mM NaCl) per wash to remove any unbound proteins and stored in TBS as a 50% suspension at 4 °C. At each time point, a sample of 10-μl suspended beads was mounted onto slides and examined under an Axio Image Z.1 microscope equipped with AxioVision (version 4.9.1.0) software (Zeiss). Images were taken with constant exposure between samples. Optical stacks were taken at 0.5 μm per optical section.

### Purification of SBP-tagged protein and biotinylated proteins using the matrices with either immobilized M18-L-DBD or M18-L-ST·SC-L-DBD

The model SBP-tagged protein was SBP-tagged β-lactamase overproduced by *B. subtilis* WB800 via secretion^9,11^. The culture supernatant of *B. subtilis* secreting the recombinant SBP-tagged β-lactamase was concentrated and dialysed against PBS to constitute the crude sample. Biotinylated BSA (12 biotin moieties/protein) was from BioVision. Biotinylated human FGF was from AcroBioSystems. Biotinylated maltose binding protein and biotinylated staphylokinase were generated by enzymatic biotinylation of MBP-AviTag (Avidity) and staphylokinase-biotinylation tag^28^ respectively by *E. coli* BirA^28^.

For all applications except where indicated, a 1-ml Sephadex G-100 column half-saturated with the respective M18-DBD fusion protein was used. This will ensure that any detached M18-DBD can easily rebind to the matrix. As the column capacity is around 708 µg for M18-L-DBD matrix and 610 µg for M18-L-ST·SC-L-DBD matrix, the M18-L-DBD column has around 354 µg of bound M18-L-DBD per ml of matrix while the M18-L-ST·SC-L-DBD column has around 305 µg of bound M18-L-ST·SC-L-DBD per ml of matrix. For each application, the contact time between the loaded sample and the matrix was 30 min. Column equilibration and wash buffers were PBS. Elution buffer was PBS with 5 mM biotin except where indicated otherwise. After sample loading, column was washed with 5–10 column volumes of wash buffer before the elution step.

### Other methods

Chemical coupling of M18 to the matrix was prepared by coupling M18 mutein purified from culture supernatant of *B. subtilis* WB800[pSAVSBPM18]^9^ onto Affi-gel 15 gel (BioRad) at a concentration of 1 mg protein/ml of gel according to the manufacturer’s instruction. Column regeneration of the M18-L-DBD matrix through extended buffer wash was detailed in Supplementary Discussion. Schematic drawing of various streptavidin, dextran-binding domain, SnoopTag and SnoopCatcher fusions with their size proportional to the sequence length was prepared using the Illustrator for Biological Sequences program^42^. The three-dimensional model of M18-L-DBD was homology modelled using the Yasara structure package^43^ and the ribbon structure was presented using Chimera^44^.

## Supplementary information

Supplementary Information

## Data Availability

Both the protein and nucleic acid sequence data for the fusions described in this manuscript are available in the Supplementary File. Since SAVSBPM18 is patented, any plasmids containing the sequence of SAVSBPM18 will be released after signing the material transfer agreement.
