# Supplementary material for: A bio-coupling approach using a dextran-binding domain to immobilize an engineered streptavidin to Sephadex for easy preparation of affinity matrix
Source: Sci Rep. 2019 Mar 4;9:3359. doi: 10.1038/s41598-019-40044-4 (PMC6399347; doi:10.1038/s41598-019-40044-4)
Supplement: Supplementary file 1 — Supplementary Information [file 41598_2019_40044_MOESM1_ESM.docx]

A bio-coupling approach using a dextran-binding domain to immobilize an engineered streptavidin to Sephadex for easy preparation of affinity matrix

Sau-Ching Wu, Chris Wang, Jonathan Chin and Sui-Lam Wong*

Department of Biological Sciences, University of Calgary, Calgary, Alberta, Canada

*Corresponding author: Department of Biological Sciences, University of Calgary, 2500 University Drive, N.W., Calgary, Alberta T2N 1N4, Canada

Phone: 403-220-5721

E-mail: [slwong@ucalgary.ca](mailto:slwong@ucalgary.ca)

**Index:**

**Supplementary Fig. S1**

**Supplementary Fig. S2**

**Supplementary Discussion**

**Supplementary Table S1**

**Supplementary sequences**

**(1) Protein and nucleic acid sequences for SAVSBPM18-Linker-DBD (M18-L-DBD)**

**(2) Protein and nucleic acid sequences for SnoopCatcher-Linker-DBD (SC-L-DBD)**

**(3) Protein and nucleic acid sequences for SAVSBPM18-Linker-SnoopTag (M18-L-ST)**

**(4) Protein and nucleic acid sequences for HisTag-SnoopCatcher-Linker-DBD(cys) [His-SC-**

**L-DBD(Cys)]**

**Supplementary Fig. S1**

**

**

**Supplementary Fig. S1. Purification of SBP-tagged β-lactamase and biotinylated proteins using the M18-L-ST·SC-L-DBD affinity matrix.** (**a**) SBP-tagged β-lactamase. S, *B. subtilis* culture supernatant containing overproduced SBP-tagged β-lactamase. Arrowhead, SBP-tagged β-lactamase. (**b**) Biotinylated BSA with 12 biotin moieties per molecule. S, loaded sample; Closed arrowhead, biotinylated BSA; Open arrowhead, Sephadex bound M18-L-ST·SC-L-DBD. (**c**) Biotinylated human FGF with 4.8 biotin moieties per molecule. S, loaded sample; Arrowhead, biotinylated human FGF. M, molecular weight markers (sizes in kDa); FT, flow-through fraction; W, wash fractions; E, elution fractions; BF, bound fraction.

**Supplementary Fig. S2**

**
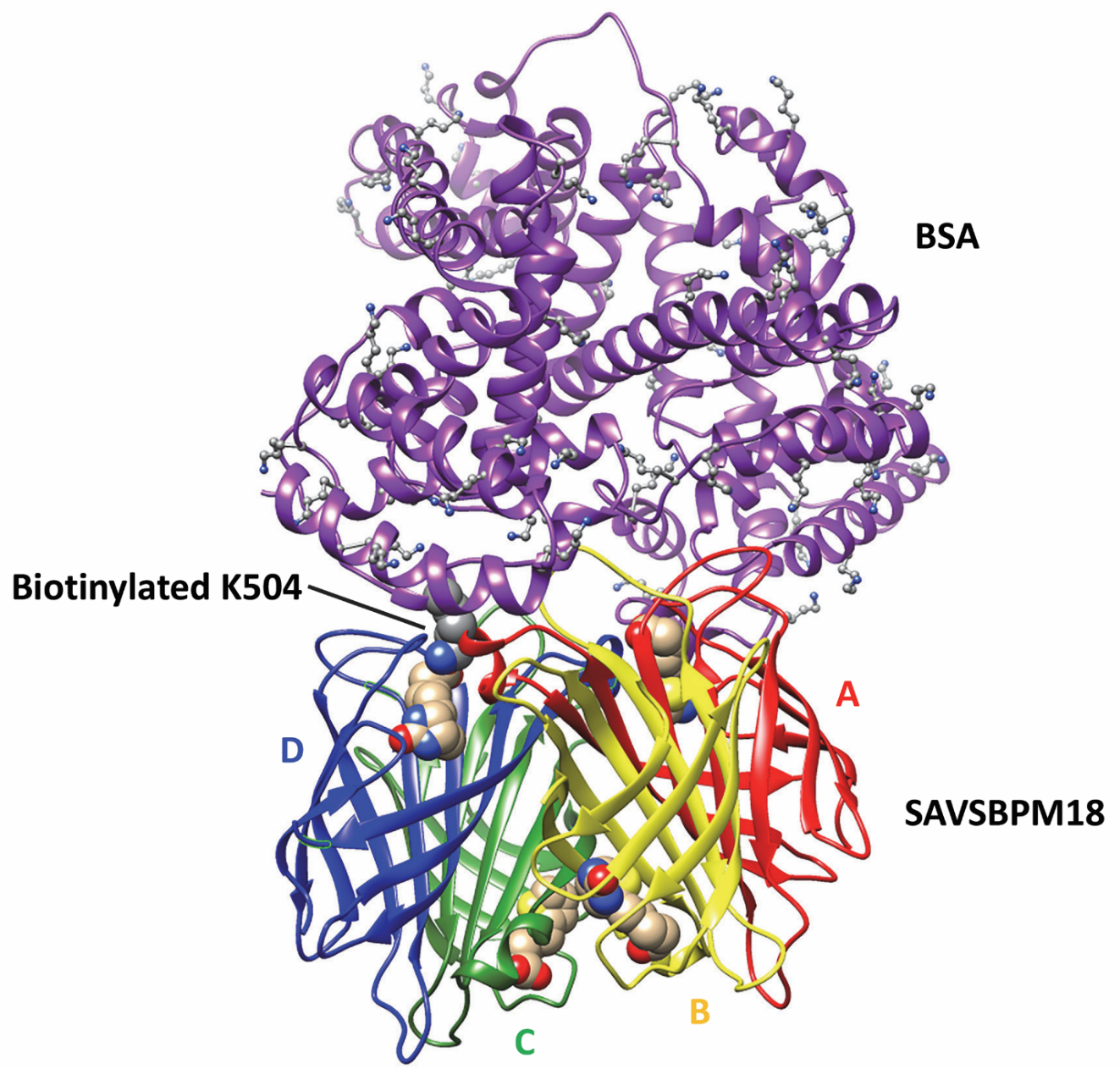
**

**Supplementary Fig. S2. Binding of a biotinylated bovine serum albumin to streptavidin.** Lysine 504, which is biotinylated, is shown in the space filled mode. Other lysine residues in BSA are shown in the ball and stick mode. Docking of BSA to streptavidin via biotinylated lysine 504 in BSA is modelled. This represents one of the possible modes for docking a biotinylated BSA to streptavidin. The four subunit units (A, B, C and D) of streptavidin are colored red, yellow, green and blue, respectively. BSA is colored purple. Biotin moieties are also shown in the space filled mode.

**Supplementary Discussion: Column regeneration efficiency**

Capturing of biotinylated BSA by M18-L-DBD immobilized Sephadex G100 column was used as a model to examine the efficiency of column regeneration. The column had 1 ml of settled beads which were half-saturated with M18-L-DBD [i.e. loaded with 354 µg (2.5 nmole) of M18-L-DBD]. Assuming one streptavidin can capture two biotinylated BSA proteins, the maximum BSA binding capacity of this column is 5 nmole or 332 µg of BSA. In each round of purification, BSA at a dosage of 400 µg was applied. The amount of biotinylated BSA in flow-through, wash and elution fractions was quantitated by Bio-Rad Protein Assay Dye Reagent. The amount captured was estimated as the balance between the amount loaded and the amount in the flow through plus wash fractions. After elution of the captured biotinylated BSA with the biotin containing elution buffer, the columns were extensively washed with at least 80 column volumes (80 ml) of wash buffer by gravity flow using a flow rate of 0.62 ml/min. As shown in the Supplementary Table S1, the column could be effectively and gently regenerated by this simple wash routine. The same column had been reused many times to capture overloading amounts of biotinylated BSA over a 5-month period. No obvious degeneration in column performance was detected. M18 and biotin interaction is a dynamic event involving both the binding and dissociation processes and the half-life of biotin to M18 has been estimated to be around 4.36 minutes^9^. Washing the column extensively using 80 column volumes of buffer in a time period greater than 2 hours can effectively remove any dissociated biotin from the matrix.

**Supplementary Table S1. Column regeneration study: Binding of biotinylated BSA to M18-L-DBD matrix**.

| Round | Amount in flow-through and wash (µg) | Amount captured (µg) | Amount eluted (µg) |
| --- | --- | --- | --- |
|  |  |  |  |
| 1 | 151.2 | 248.8 | 223.9 |
| 2 | 144.5 | 255.5 | 235.1 |
| 3 | 149.6 | 250.4 | 232.9 |
| 4 | 148.2 | 251.8 | 234.2 |
|  |  |  |  |
| Average | 148.4 | 251.6 | 231.5 |
| SD | 2.9 | 2.9 | 5.2 |

A 1-ml M18-L-DBD immobilized Sephadex-G100 column (with 2.5 nmoles of M18-L-DBD immobilized) was overloaded with 400 µg biotinylated BSA in each round. The column was then washed to remove any unbound biotinylated BSA, followed by elution with biotin and the extended wash to regenerate the column. The regenerated column then went through three more cycles of loading, washing, elution and regeneration steps to monitor the column regeneration efficiency. Biotinylated BSA in different fractions was quantitated by Bio-Rad Protein Assay Dye Reagent.

**Supplementary sequences**

**Sequence 1:** **SAVSBPM18-Linker-DBD (M18-L-DBD)**

**MDPSKDSKAQVSAAEAGITGTWYNQLGATFIVTAGADGALTGTYESAVTN 50**

**AESRYVLTGRYDSAPATDGSGTALGWTVAWKNNYRNAHSATTWSGQYVGG 100**

**AEARINTQWLLTSGTTEANAWKSTLVGHDTFTKVKPSAASIDAAKKAGVN 150**

**NGNPLDAVQQSPGSGPGTAGGTSPTSGSTPEGDPSTSGNQYYQLADGKYM 200**

**LLDDSGRAKTGFVLQDGVLRYFDQNGEQVKDAIIVDPDTNLSYYFNATQG 250**

**VAVKNDYFEYQGNWYLTDANYQLIKGFKAVDDSLQHFDEVTGVQTKDSAL 300**

**ISAQGKVYQFDNNGNAVSAPSNPSPSGSSTGAPA**

**A C-terminal sequence (black) is added to the C-terminal end of DBD.**

**Nucleic acid sequence for M18-L-DBD**

**ATGGA CCCGAGCAAA GATTCTAAAG**

**CACAAGTATC TGCTGCAGAA GCGGGCATTA CGGGCACGTG GTATAATCAG**

**CTGGGCGCTA CGTTTATTGT TACGGCCGGC GCAGATGGAG CTCTGACGGG**

**CACGTATGAA AGCGCGGTTA CAAATGCAGA ATCTAGATAC GTTCTTACAG**

**GAAGATATGA TTCTGCACCT GCAACAGATG GATCCGGCAC GGCACTGGGC**

**TGGACAGTTG CATGGAAAAA CAATTATCGC AACGCACATA GCGCCACGAC**

**GTGGTCTGGC CAATATGTTG GCGGTGCAGA AGCACGCATT AACACACAGT**

**GGCTTCTGAC GTCCGGAACA ACAGAAGCAA ATGCATGGAA AAGTACTCTT**

**GTTGGACATG ATACATTTAC AAAAGTTAAA CCTAGCGCAG CATCTATCGA**

**TGCAGCGAAA AAAGCAGGAG TTAACAATGG TAACCCTCTG GATGCAGTTC**

**AACAATCACC TGGTAGCGGA CCGGGAACGG CCGGGGGGAC TAGTCCGACG**

**TCAGGCTCGA CACCTGAGGG AGACCCGAGC ACTAGTGGAA ACCAGTACTA**

**TCAGCTGGCT GATGGCAAGT ACATGCTTCT TGATGACAGT GGCCGCGCGA**

**AAACAGGTTT TGTCCTTCAG GATGGGGTCT TAAGATATTT TGACCAAAAC**

**GGTGAACAAG TCAAAGATGC AATAATTGTC GACCCAGATA CGAATCTGAG**

**CTATTATTTT AATGCGACAC AGGGTGTTGC GGTAAAGAAC GATTACTTTG**

**GGTTTTAAAG CAGTTGACGA TAGTCTTCAG CATTTTGATG AGGTGACCGG**

**TGTGCAGACA AAGGATTCTG CTCTGATATC AGCACAAGGG AAAGTTTACC**

**AGTTTGACAA CAATGGCAAC GCGGTCAGCG CTCCTTCGAA TCCGTCTCCG**

**TCTGGATCAA GTACTGGCGC GCCGGCTTAA**

**Sequence 2: SnoopCatcher-Linker-DBD (SC-L-DBD)**

**MTSDSGQGKPLRGAVFSLQKQHPDYPDIYGAIDQNGTYQNVRTGEDGKLT 50**

**FKNLSDGKYRLFENSEPAGYKPVQNKPIVAFQIVNGEVRDVTSIVPQDIP 100**

**ATYEFTNGKHYITNEPIPPKGAPSSNGPSPGSGPGTAGGTSPTSGSTPEG 150**

**DPSTSGNQYYQLADGKYMLLDDSGRAKTGFVLQDGVLRYFDQNGEQVKDA 200**

**IIVDPDTNLSYYFNATQGVAVKNDYFEYQGNWYLTDANYQLIKGFKAVDD 250**

**SLQHFDEVTGVQTKDSALISAQGKVYQFDNNGNAVSAPSNPSPSGSSTGA 300**

**PA**

**Short N-terminal (red) and C-terminal (black) sequences are added to N- and C-termini, respectively.**

**Nucleic acid sequence for SC-L-DBD**

**ATGAC TAGTGACAGC GGTCAAGGTA**

**AACCGCTGCG CGGCGCCGTG TTCAGCTTAC AGAAGCAACA TCCGGATTAT**

**CCGGACATCT ATGGCGCAAT TGATCAGAAT GGCACCTACC AGAACGTTCG**

**TACGGGTGAA GATGGCAAAC TGACCTTTAA AAATCTGAGC GACGGCAAAT**

**ATCGTCTGTT CGAGAACAGC GAACCGGCCG GCTACAAACC TGTGCAGAAC**

**AAACCGATTG TTGCCTTTCA AATTGTTAAT GGCGAAGTGC GCGACGTCAC**

**GAGCATTGTG CCTCAGGATA TTCCGGCGAC CTACGAGTTT ACCAACGGTA**

**AACATTATAT CACCAACGAA CCGATTCCGC CGAAAGGCGC GCCGTCTTCG**

**AACGGTCCGT CACCTGGTAG CGGACCGGGA ACGGCCGGGG GGACTAGTCC**

**GACGTCAGGC TCGACACCTG AGGGAGACCC GAGCACTAGT GGAAACCAGT**

**ACTATCAGCT GGCTGATGGC AAGTACATGC TTCTTGATGA CAGTGGCCGC**

**GCGAAAACAG GTTTTGTCCT TCAGGATGGG GTCTTAAGAT ATTTTGACCA**

**AAACGGTGAA CAAGTCAAAG ATGCAATAAT TGTCGACCCA GATACGAATC**

**TGAGCTATTA TTTTAATGCG ACACAGGGTG TTGCGGTAAA GAACGATTAC**

**TTTGAATATC AGGGTAACTG GTATCTCACT GATGCTAACT ACCAGTTAAT**

**TAAAGGTTTT AAAGCAGTTG ACGATAGTCT TCAGCATTTT GATGAGGTGA**

**CCGGTGTGCA GACAAAGGAT TCTGCTCTGA TATCAGCACA AGGGAAAGTT**

**TACCAGTTTG ACAACAATGG CAACGCGGTC AGCGCTCCTT CGAATCCGTC**

**TCCGTCTGGA TCAAGTACTG GCGCGCCGGC TTAA**

**Sequence 3: SAVSBPM18-Linker-SnoopTag (M18-L-ST)**

**MDPSKDSKAQVSAAEAGITGTWYNQLGATFIVTAGADGALTGTYESAVTN 50**

**AESRYVLTGRYDSAPATDGSGTALGWTVAWKNNYRNAHSATTWSGQYVGG 100**

**AEARINTQWLLTSGTTEANAWKSTLVGHDTFTKVKPSAASIDAAKKAGVN 150**

**NGNPLDAVQQSPGSGTAGGTSPTSGSTPEGDPSTSGGPSGGGGSGKLGDI 200**

**EFIKVNKGS**

**A methionine residue and a two-residue sequence (black) are added to N- and C-termini, respectively.**

**Nucleic acid sequence for M18-L-ST**

**ATGGA CCCGAGCAAA GATTCTAAAG**

**CACAAGTATC TGCTGCAGAA GCGGGCATTA CGGGCACGTG GTATAATCAG**

**CTGGGCGCTA CGTTTATTGT TACGGCCGGC GCAGATGGAG CTCTGACGGG**

**CACGTATGAA AGCGCGGTTA CAAATGCAGA ATCTCGCTAC GTTCTTACAG**

**GAAGATATGA TTCTGCACCT GCAACAGATG GATCCGGCAC GGCACTGGGC**

**TGGACAGTTG CATGGAAAAA CAATTATCGC AACGCACATA GCGCCACGAC**

**GTGGTCTGGC CAATATGTTG GCGGTGCAGA AGCACGCATT AACACACAGT**

**GGCTTCTGAC GTCCGGAACA ACAGAAGCAA ATGCATGGAA AAGTACTCTT**

**GTTGGACATG ATACATTTAC AAAAGTTAAA CCTAGCGCAG CATCTATCGA**

**TGCAGCGAAA AAAGCAGGAG TTAACAATGG TAACCCTCTG GATGCAGTTC**

**AACAATCACC TGGTAGCGGA ACGGCCGGGG GGACTAGTCC GACGTCAGGC**

**TCGACACCTG AGGGAGACCC GAGCACTAGT GGCGGTCCGT CAGGTGGCGG**

**TGGCAGCGGG AAACTGGGCG ATATTGAATT TATTAAAGTG AACAAAGGCA**

**GCTAA**

**Sequence 4: HisTag-SnoopCatcher-Linker-DBD(cys) [His-SC-L-DBD(Cys)]**

**MGHHHHHHHHHHSSGHIDDDDKHMTSDSGQGKPLRGAVFSLQKQHPDYPD 50**

**IYGAIDQNGTYQNVRTGEDGKLTFKNLSDGKYRLFENSEPAGYKPVQNKP 100**

**IVAFQIVNGEVRDVTSIVPQDIPATYEFTNGKHYITNEPIPPKGAPSSNG 150**

**PSPGSGPGTAGGTSPTSGSTPEGDPSTSGNQYYQLADGKYMLLDDSGRAK 200**

**TGFVLQDGVLRYFDQNGEQVKDAIIVDPDTNLSYYFNATQGVAVKNDYFE 250**

**YQGNWYLTDANYQLIKGFKAVDDSLQHFDEVTGVQTKDSALISAQGKVYQ 300**

**FDNNGNAVSAPSTPEGCGTPSNTAGAPAA**

**A sequence (black) containing a single cysteine residue is added to the C-terminal end.**

**Nucleic acid sequence for His-SC-L-DBD(Cys)**

**ATGAC TAGTGACAGC GGTCAAGGTA**

**AACCGCTGCG CGGCGCCGTG TTCAGCTTAC AGAAGCAACA TCCGGATTAT**

**CCGGACATCT ATGGCGCAAT TGATCAGAAT GGCACCTACC AGAACGTTCG**

**TACGGGTGAA GATGGCAAAC TGACCTTTAA AAATCTGAGC GACGGCAAAT**

**ATCGTCTGTT CGAGAACAGC GAACCGGCCG GCTACAAACC TGTGCAGAAC**

**AAACCGATTG TTGCCTTTCA AATTGTTAAT GGCGAAGTGC GCGACGTCAC**

**GAGCATTGTG CCTCAGGATA TTCCGGCGAC CTACGAGTTT ACCAACGGTA**

**AACATTATAT CACCAACGAA CCGATTCCGC CGAAAGGCGC GCCGTCTTCG**

**AACGGTCCGT CACCTGGTAG CGGACCGGGA ACGGCCGGGG GGACTAGTCC**

**GACGTCAGGC TCGACACCTG AGGGAGACCC GAGCACTAGT GGAAACCAGT**

**ACTATCAGCT GGCTGATGGC AAGTACATGC TTCTTGATGA CAGTGGCCGC**

**GCGAAAACAG GTTTTGTCCT TCAGGATGGG GTCTTAAGAT ATTTTGACCA**

**AAACGGTGAA CAAGTCAAAG ATGCAATAAT TGTCGACCCA GATACGAATC**

**TGAGCTATTA TTTTAATGCG ACACAGGGTG TTGCGGTAAA GAACGATTAC**

**TTTGAATATC AGGGTAACTG GTATCTCACT GATGCTAACT ACCAGTTAAT**

**TAAAGGTTTT AAAGCAGTTG ACGATAGTCT TCAGCATTTT GATGAGGTGA**

**CCGGTGTGCA GACAAAGGAT TCTGCTCTGA TTTCAGCACA AGGGAAAGTT**

**TACCAGTTTG ACAACAATGG CAACGCGGTC AGCGCTCCTT CGACACCTGA**

**GGGATGCGGC ACTCCTTCGA ATACCGCGGG CGCGCCGGCT GCATAA**
